# Infection of Human Retinal Pigment Epithelium with *Chlamydia trachomatis*

**DOI:** 10.1371/journal.pone.0141754

**Published:** 2015-11-04

**Authors:** Ernest Boiko, Dmitrii Maltsev, Alevtina Savicheva, Kira Shalepo, Tatyana Khusnutdinova, Alexei Pozniak, Igor Kvetnoi, Viktoria Polyakova, Alexei Suetov

**Affiliations:** 1 Department of Ophthalmology, Military Medical Academy, St Petersburg, Russia; 2 St. Petersburg Branch of the Academician S. Fyodorov IRTC “Eye Microsurgery”, St. Petersburg, Russia; 3 Laboratory of Microbiology, D.O. Ott Research Institute of Obstetrics and Gynaecology, St. Petersburg, Russia; 4 Pathology Department, D.O. Ott Research Institute of Obstetrics and Gynaecology, St. Petersburg, Russia; Rutgers University, UNITED STATES

## Abstract

**Purpose:**

Little is known about the susceptibility of posterior segment tissues, particularly the human retinal pigment epithelium (hRPE), to *Chlamydia trachomatis*. The purpose of the study was to investigate the possibility of infecting the hRPE with *Chlamydia trachomatis*, and to examine the infectivity of different *Chlamydia trachomatis* clinical isolates for hRPE cells and the hRPE cell response to the infection.

**Methods:**

Cultured hRPE and McCoy cells were inoculated with eight *Chlamydia trachomatis* (serovar E) clinical isolates at multiplicity of infection (MOI) of 2.0 or 0.3. To detect *Chlamydia trachomatis*, samples were stained immunohistochemically with anti-major outer membrane protein antibodies at 24h, 48h, and 72h postinoculation (PI). The changes in the expression of signaling molecules and proteins of cytoskeleton and extracellular matrix in hRPE cells were examined immunohistochemically.

**Results:**

All eight clinical isolates demonstrated ability to infect hRPE cells. At equal MOI of 0.3, the infectivity of *Chlamydia trachomatis* clinical isolates for RPE culture was found to be at least as high as that for McCoy cell culture. At 24h PI, the percentage of inclusion-containing cells varied from 1.5 ± 0.52 to 14.6 ± 3.3% in hRPE cell culture infected at MOI of 2.0 against 0.37 ± 0.34 to 8.9 ± 0.2% in McCoy cell culture infected at MOI of 0.3. Collagen type I, collagen type IV, basic fibroblast growth factor, transforming growth factor-beta and interleukin–8 expression at 48h PI were maximally increased, by 2.1-, 1.3-, 1.5-, 1.5- and 1.6-fold, respectively, in the *Chlamydia trachomatis*-infected compared with control hRPE cell culture specimens (*P* < 0.05).

**Conclusions:**

This study, for the first time, proved the possibility of infecting hRPE cultured cells with *Chlamydia trachomatis*, which leads to proproliferative and proinflammatory changes in the expression of signaling molecules and extracellular matrix components.

## Introduction

Retinal pigment epithelium (RPE) is critically important for maintaining the structural and functional integrity of the retina and choriocapillaris. Changes in the RPE play a key role in the pathogenesis of various posterior segment disorders, including age-related macular degeneration and proliferative vitreoretinopathy.

Numerous factors (including oxidative stress, inflammation, genetic specificity, etc.) condition the modulation of biological characteristics of RPE cells, leading to changes in their functional activity. Two of the most significant factors influencing the RPE are proinflammatory stimuli and signaling molecules participating in inflammation [1, 2]. The marked influence of these molecules on the biological characteristics of the human RPE (hRPE) has been demonstrated *in vitro*. This involves the increase in complement expression by RPE cells stimulated by activated macrophages [1] and an enhanced RPE cell migration and proliferative activity stimulated by epidermal (EGF), platelet-derived (PDGF) and basic fibroblast growth factors (bFGF).[2,3]

Infection is one of the main causes of inflammation that develops either due to direct cell infection, or through immune mechanisms. Stimulation of hRPE with *C*. *pneumoniae* antigen [4] and the proinflammatory effect of a combination of virulence factors of *S*. *aureus* on the expression of cytokines in RPE cells [5] have been demonstrated *in vitro*. *In vivo*, direct infection of RPE with Varizella Zoster and Cytomegalovirus has been demonstrated in human autopsy specimens [6, 7] and in experimental animal studies [8]. Several works have shown the possibility of *in vitro* damage to cultured hRPE cells by neurotropic viruses that can cause ocular involvement (particularly, retinitis and chorioretinitis) in humans [9–11]. Most of the works on infectious RPE damage, however, are related to viral infections, whereas the effect of bacterial infections has been poorly investigated.

In our previous work, we have demonstrated infection of the RPE with the obligate intracellular human bacterial pathogen, *Chlamydia trachomatis*, following ocular inoculation in rabbits [12]. Yet, the possibility of infecting the hRPE with *Chlamydia trachomatis* and the pathogenetic significance of such an infectious process remain unknown. Our current aim, therefore, was to investigate the possibility of infecting the hRPE with *Chlamydia trachomatis*, and to examine both the infectivity of different clinical isolates of the pathogen for hRPE cells and the response of hRPE cells to the infection.

## Materials and Methods

### Ethics statement

The study was conducted in compliance with the Russian Bioethical law (Law no. 4180-I of December 22, 1994, as revised on November 29, 2007) and approved by the local Ethics Committee of the Military Medical Academy. The protocol regarding use of donor eyes adhered to the provisions of the Declaration of Helsinki for research involving human tissue.

i) *C*. *trachomatis* clinical isolates: samples of *C*. *trachomatis* clinical isolates (serovar E) were obtained from patients of the Ott Research Institute of Obstetrics and Gynaecology during treatment and diagnostic manipulations. Written consent for sampling of clinical isolates for research purpose was obtained as approved by the Ethics Committees of the Ott Research Institute of Obstetrics and Gynaecology and Military Medical Academy.

ii) globes from deceased donors: prior to harvesting the organ for donation, the legal next of kin of each deceased donor gave a written informed consent for use of the globe for transplantation and research (as part of the overall protocol and consent for organ donation).

### Cell Cultures

#### RPE

hRPE cultures were obtained from 6 cadaveric eyes within 36 hours after death, and after the cornea had been removed for transplantation surgery, using a technique described earlier [10]. Briefly, after external tissues were removed, the globe was rinsed with 70% ethanol and twice washed with sterile Hanks’ balanced salt solution (HBSS) (GIBCO-BRL, Grand Island, NY). The anterior segment, vitreous and retina were removed. The eyecup was incubated with 0.2 mg/ml collagenase type 1 (GIBCO-BRL) for 40 minutes at 37°C and 5% CO_2_. One point five to 1.8 × 10^6^ cells per donor eye were collected by gentle pipetting, and the resulting suspension was centrifuged at 300g for 4 min. After removal of supernatant, cells were resuspended in DMEM/F12 medium (Biolot, St.Petersburg, Russia) supplemented with streptomycin, penicillin and amphotericin (GIBCO-BRL) and 10% bovine serum albumine (BSA) (GIBCO-BRL), and seeded into culture flasks. Cells were passaged at a 1:3 ratio when 70–80% confluent; usually, each passage was accompanied by a three- to four-fold increase in cell numbers. The hRPE used in all experiments were from passage 4 to 5. The purity of the cell line was confirmed by detection of cytokeratin 8/18 expression (Table 1).

**Table 1:** Primary Antibodies Used to Detect *C*. *trachomatis* Antigens, Signaling, Cytoskeleton and Extracellular Matrix Molecules. *MOMP*, major outer membrane protein; *VEGF*, vascular endothelial growth factor; *TNF–α*, tumor necrosis factors–α; *α-SMA*, smooth muscle actin; *MMP–9*, matrix metalloproteinase 9; *bFGF*, basic fibroblast growth factor; *TGF–β*, transforming growth factor β; *RFU*, ready for use

Antigens	Host	Type	Number	Dilution	Manufacturer
MOMP *C*.* trachomatis*	Mouse	Polyclonal	AM088-5M	RFU	BioGenex, Mountainview, CA
MOMP *C*.* trachomatis* (fluorescein-isothiocyanate-conjugated)	Chicken	Polyclonal	039487	RFU	LabDiagnostic, Moscow, Russia
VEGF	Mouse	Monoclonal	M7273	1:100	DAKO, Santa Barbara, CA
Collagen type IV	Mouse	Monoclonal	M0785	1:50	DAKO
Interleukin-8	Rabbit	Polyclonal	ab7747	1:100	ABCAM, Cambridge, MA
TNF–α	Rabbit	Polyclonal	ab6671	1:50	ABCAM
α-SMA	Mouse	Monoclonal	LS-C95386	RFU	LSBio, Seattle, WA
MMP-9	Mouse	Monoclonal	NCL-MMP9-439	1:40	Novocastra, Newcastle, UK
Collagen type I	Rabbit	Polyclonal	ab34710	1:50	ABCAM
bFGF	Mouse	Monoclonal	610073	1:100	BD Transduction, San Jose, CA
TGF–β	Mouse	Monoclonal	ab64715	1:50	ABCAM
E-cadherin	Mouse	Monoclonal	CM 170 C	1:100	BioCare, Concord, CA
CD105	Rabbit	Monoclonal	NCL-CD105	1:100	Novocastra
Cytokeratin 8/18	Mouse	Monoclonal	MS-743-S	1:50	Thermo Scientific, Rockford, IL

To perform the experiments, cells were transferred to 96-well flat-bottom plates (5×10^3^ cells per well by the time of infection) five days preinoculation.

#### McCoy

McCoy cell culture was provided by the Cell Culture Bank of the Ott Institute of Obstetrics and Gynaecology (St. Petersburg, Russia), and grown to confluence in DMEM/F12 containing gentamicin, amphotericin, and 10% BSA. Twenty-four hours preinoculation, after being trypsinized, cells were seeded into 96-well plates (3×10^4^ cells per well by the time of infection).

### Pathogen and Inoculation

Eight *Chlamydia trachomatis* clinical isolates (serovar E) of patients from the Ott Institute of Obstetrics and Gynaecology were selected for inoculation of cell cultures. Viability of the pathogen, its infectivity for cell cultures, and *Chlamydia trachomatis* doses were tested with McCoy cell culture. Infected McCoy cell monolayers were detached by scraping and disrupted by sterile glass beads to release elementary bodies (EBs). Cell debris was removed by centrifugation (500g, 15 min). Each non-control well of a 96-well plate received 100 μL of cell lysate containing a fixed number of inclusion forming units of a *Chlamydia trachomatis* clinical isolate (Table 2). Each control well of the same plate received 100 μL of uninfected cell lysate as a mock infection control. The plates were then centrifuged at 2400 rpm for 60 min at 37°C, and the medium was completely changed with the same medium (DMEM/F12, 10% BSA) to which 1 mg/L cycloheximide (Acros Organics, Geel, Belgium) was added only in the experiments for the assessment of the infectivity of the pathogen. To verify the completion of C. trachomatis life cycle, clinical isolate No. 24032 was repassaged from individual RPE cell culture samples to RPE and McCoy cell cultures using the technique described above 72 hpi. Intracellular inclusions were visualized by direct immune fluorescence 48 hours after repassage. For immunohistochemistry and direct immunofluorescent staining, the culture medium was removed from plate wells, and the cells were washed twice with HBSS and fixed at 24h, 48h, and 72h postinoculation.

**Table 2:** Clinical Isolates of *C*. *trachomatis*, the Doses Used to Infect Cell Cultures and Multiplicities of Infection Corresponding to the Doses and to the Isolates. *IFU*, inclusion forming units; *MOI*, multiplicity of infection

Сlinical isolate number	1609	3438	24032	27692	5017	27644	685	2556
IFU for RPE	10^4^	10^4^	10^4^	10^4^	10^4^	1.7×10^3^	1.7×10^3^	1.7×10^3^
IFU for McCoy	10^4^	10^4^	10^4^	10^4^	10^4^	10^4^	10^4^	10^4^
MOI for RPE	2.0	2.0	2.0	2.0	2.0	0.3	0.3	0.3
MOI for McCoy	0.3	0.3	0.3	0.3	0.3	0.3	0.3	0.3

### Immunohistochemistry and Immunofluorescence Detection, and Assessment of the Infectivity of the Pathogen

#### Immunohistochemistry

For immunohistochemical staining, cells were fixed in 4% paraformaldehyde for 15 minutes at 20°C, permeabilized with 0.01% Triton X-100 for 15 minutes and rinsed 3 times with PBS for 5 minutes each time. They were then blocked for endogenous peroxidase using 3% H_2_O_2_ in PBS for 15 minutes and again rinsed 3 times with PBS for 5 minutes each time. This was followed by incubation for 15 minutes in 1% BSA. The cells were then exposed overnight to a primary mouse anti-major outer membrane protein antibody (Table 1). The primary antibodies were detected by incubating the cells with a goat anti-mouse/anti-rabbit horseradish peroxidase-conjugated secondary antibody (DAKO, Santa Barbara, CA) for 30 min at 20°C. Diaminobenzidine (DAKO) and hematoxylin were used as the chromogen and counterstain, respectively.

#### Direct immunofluorescent staining

For direct immunofluorescent staining, cells were fixed in 4% paraformaldehyde for 15 minutes at 20°C and rinsed 3 times with PBS for 5 minutes each time. The cells were then incubated with fluorescein-isothiocyanate-conjugated antibody to *Chlamydia trachomatis* major outer membrane protein (Table 1) for 30 min at 37°C in a dark, humidified chamber; Evans blue was used as a counterstain. After being washed twice in PBS, dried, and coverslipped with PBS/glycerol, the slides were examined using an Axioplan 2 fluorescence microscope (Carl Zeiss, Jena, Germany) (excitation wavelength, 490 nm).

#### Assessment of the Infectivity of the Pathogen

At each examination time-point, the percentage of inclusion-containing cells (representing the infectivity of a *Chlamydia trachomatis* clinical isolate) and the morphology of intracellular inclusions were assessed both in immunohistochemistry and in direct immunofluorescent staining. The percentage was averaged over 10 fields of view per slide based on the results of two repeated experiments (with hRPE culture from one donor). For each clinical isolate, the percentage of inclusion-containing cells was determined as an average of two repeated experiments.

### Immunohisctochemistry of Signaling Molecules and Proteins of Cytoskeleton and Extracellular Matrix

To examine the post-inoculation changes in the expression of signaling molecules, cytoskeleton and extracellular matrix (ECM) components in RPE cells, cells were plated in 96-well plates (3×10^3^ cells per well) in DMEM:F12 medium supplemented with 10% BSA and without cycloheximide. After 72 hours, the medium was changed to DMEM:F12 supplemented with 1% BSA and without cycloheximide. Additionally, non-control wells were infected as described above with clinical isolates Nos.24032 or 27692 (5×10^3^ inclusion forming units per well) that had exhibited the highest infectivity. After 48 hours, the slides were fixed in 4% paraformaldehyde and stained with antibodies (Table 1) as described above, but without hematoxylin counterstaining.

Optical density of staining was analyzed using Image J (NIH, Bethesda, MD) as follows: (1) Digital color images were processed using the “Subtract Background” function and (2) converted to 32-bit greyscale images; (3) The “Measure” function was used to determine mean background pixel values for each of 5 circular cell-free areas (with each circle having a diameter of up to 10 pixels) within a well; (4) Mean pixel values were determined for each of 5 spaced circular areas (with each circle having a diameter of 200 pixels and involving no cell-free subareas) showing a homogeneous cell distribution; (5) The data were transferred to Microsoft Excel 2010 (Microsoft Corp., Redmond, WA). Then the mean background pixel value (for all 5 circular cell-free areas) and the mean pixel value (for all 5 spaced circular areas showing a homogeneous cell distribution) were calculated. Thereafter, the difference of these mean values was calculated, with the results normalized to those of control.

Such an approach enabled us to avoid incorporation of areas showing a heterogeneous cell distribution or cell-free areas into the analysis, and to compensate for the different background colors.

*Chlamydia trachomatis* infection of cultured cells was confirmed and the infection level was quantified by means of immunohistochemical staining as described above. The experiment was repeated twice with cells at the same passage from different donor eyes. The optical density value was determined as an average of two repeated experiments.

### Statistical Analysis

Statistical analysis was performed with a commercial spreadsheet program (Excel; Microsoft Corporation, Redmond, WA) and Statistica 6.0 software (StatSoft, Inc., Tulsa, OK). Statistical significance of differences (1) in the infectivity of the same clinical isolate for different host cell cultures and (2) in optical density of immunohistochemical staining between various clinical isolates was determined by a one-way ANOVA-test with Bonferroni post hoc test. *P* values below 0.05 were considered statistically significant.

## Results

### Cell Culture Susceptibility and Dose-dependent Susceptibility to Chlamydia trachomatis Infection

**Fig 1:**
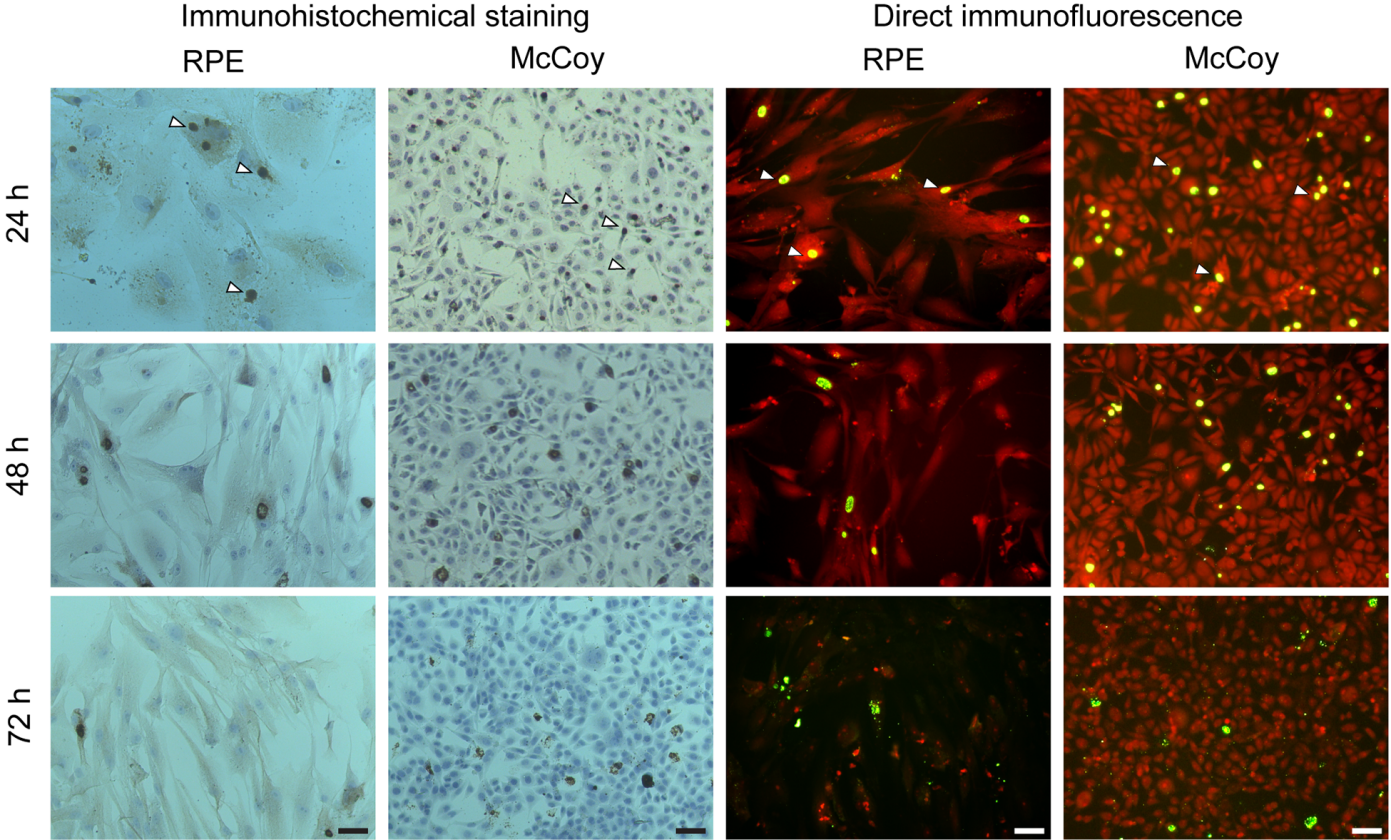
Immunohistochemistry and direct immunofluorescent staining of hRPE and McCoy cell cultures at different time-points postinoculation with *Chlamydia trachomatis* (clinical isolate No.24032). In both types of cultures, both techniques reveal highly immunoreactive inclusions (arrowheads). Twenty-four to 72 h postinoculation, a reduction in the number of intracellular inclusions is observed due to the release of a new generation of EBs to the extracellular environment. Scale bar: 50 μm.

**Fig 2:**
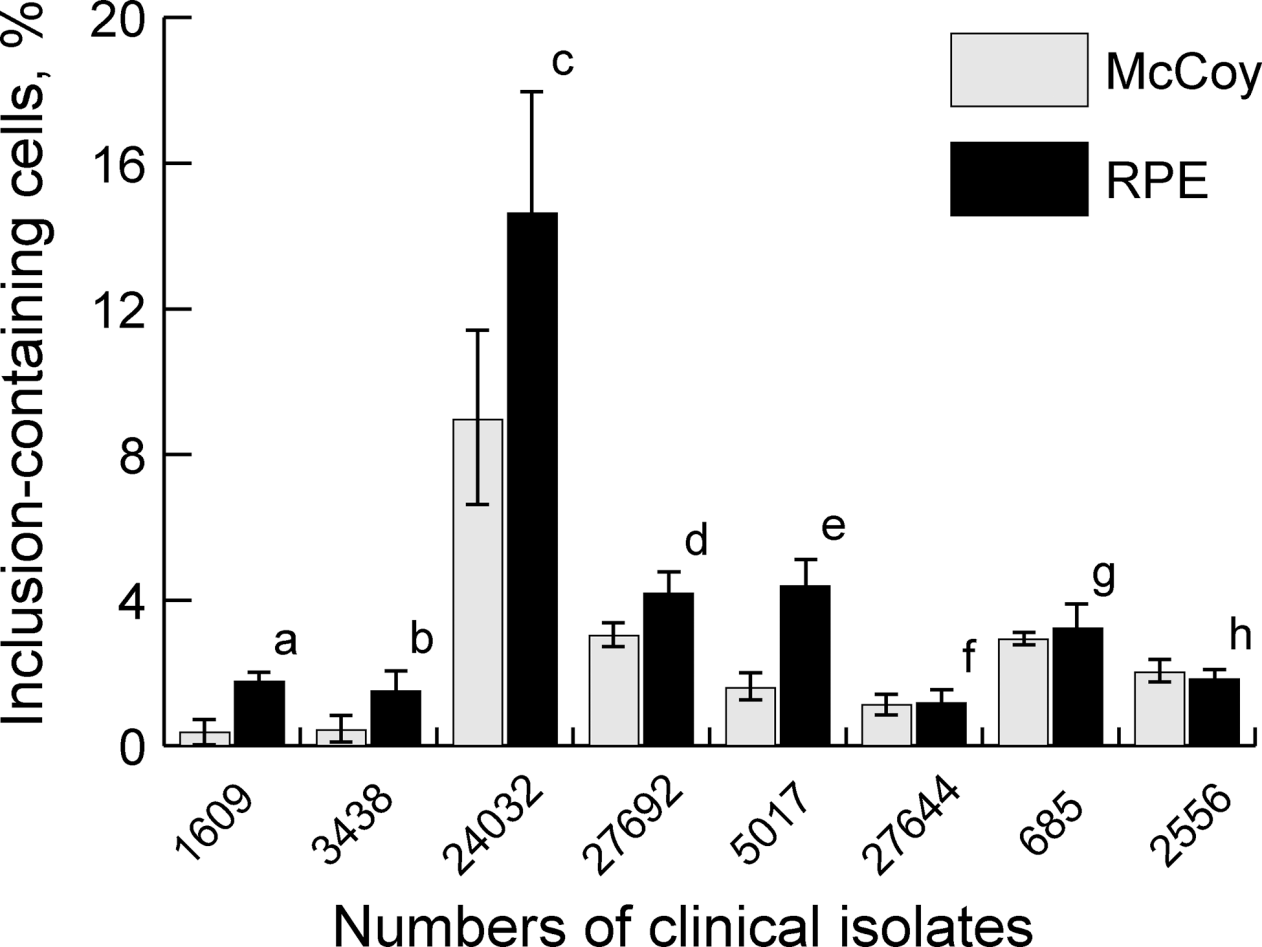
Percentage of inclusion-containing cells in hRPE and McCoy cell cultures following inoculation with different *Chlamydia trachomatis* clinical isolates. a, *P* = 0.001; b, *P* = 0.0002; c, *P* = 0.01; d, *P* = 0.039; e, *P* = 0.018; f, *P* = 0.76; g, *P* = 0.18; h, p = 0.22

In all non-control samples of RPE and McCoy cell cultures, the pathogen was detected both by immunohistochemistry and by direct immunofluorescent staining (Fig 1). Twenty-four hours postinoculation (hpi), clinical isolates (at equal multiplicity of infection (MOI) of 2.0) demonstrated a wide variation in infectivity, with the percentage of RPE culture inclusion-containing cells ranging from 1.5±0.52% (isolate No.3438) to 14.6±3.3% (isolate No.24032), whereas that of McCoy culture cells infected at equal MOI of 2.0 was 0.37±0.34% (isolate No.1609) to 8.9±2.4% (isolate No.24032) (Fig 2). Twenty-four hpi, the variation in the percentage of RPE culture inclusion-containing cells among five clinical isolates used for infection at MOI 2.0 was almost 10-fold (1.5–14.6%). All the five clinical isolates used at a higher MOI for infection of RPE cells than for infection of McCoy cells showed a significantly higher (*P*<0.05) percentage of inclusion-containing cells for hRPE cell culture than for McCoy cell culture. At equal MOI of 0.3, no statistically significant difference in the percentage of inclusion-containing cells was found between McCoy cells and RPR cells.

### Chlamydia trachomatis Life Cycle in Vitro

In the hRPE culture, like in the control culture, the maximal number of intracellular inclusions (mostly small and medium size, perinuclear inclusions) of *Chlamydia trachomatis* was observed 24 hours hpi (Fig 1). A few isolated elementary bodies (EBs) (most likely, from the inoculating material) were found on the surface of some cells.

**Fig 3:**
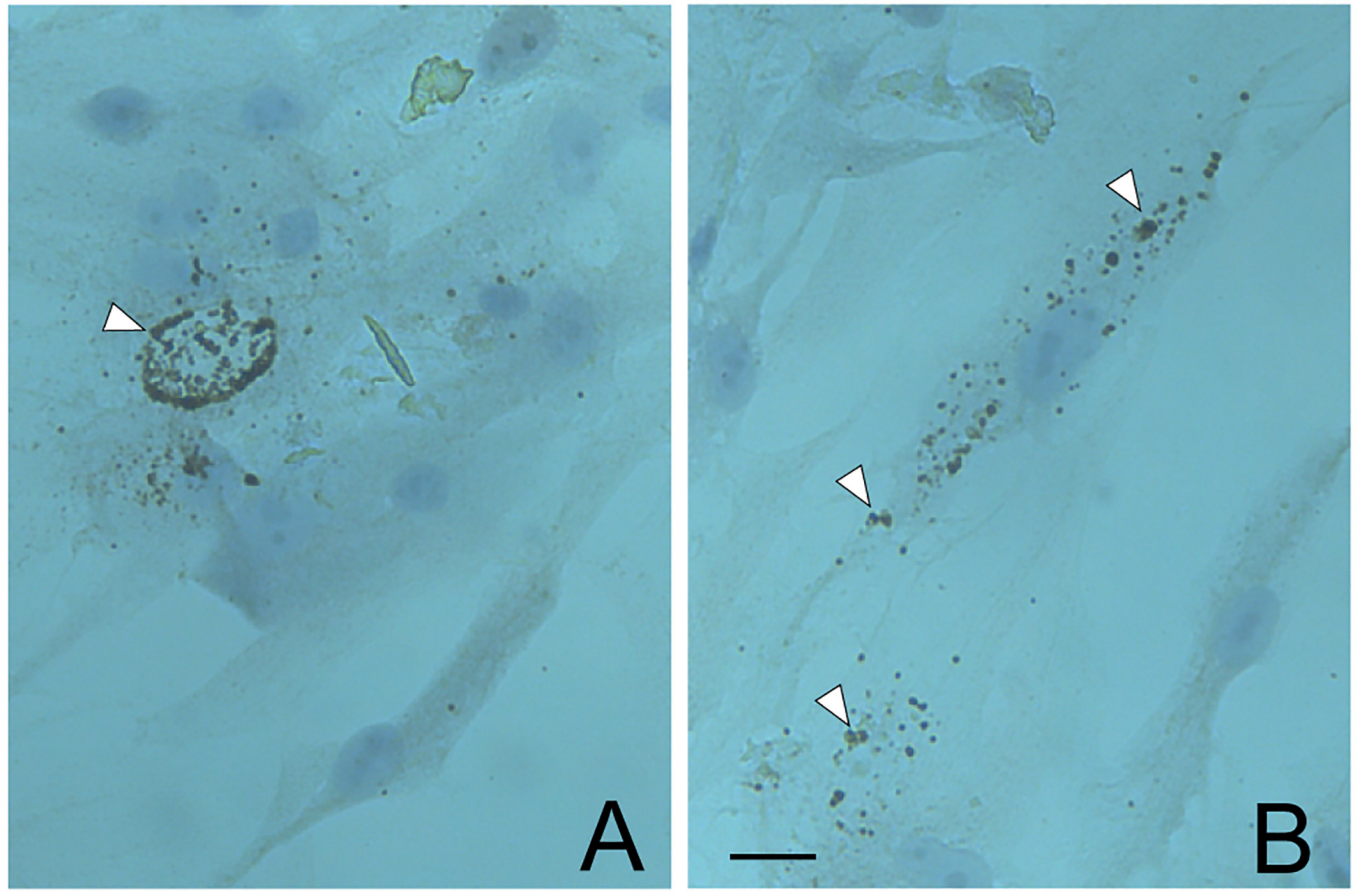
Immunohistochemistry micrographs of hRPE cell culture at 72 hours postinoculation with *Chlamydia trachomatis* clinical isolate No.24032. (A) Intracellular inclusions (arrowhead) exhibiting weak immune responsiveness (due to release of EBs to extracellular environment). (B) Numerous EBs (arrowheads) in microscopy projection of cells without inclusions. Scale bar: 10 μm.

**Fig 4:**
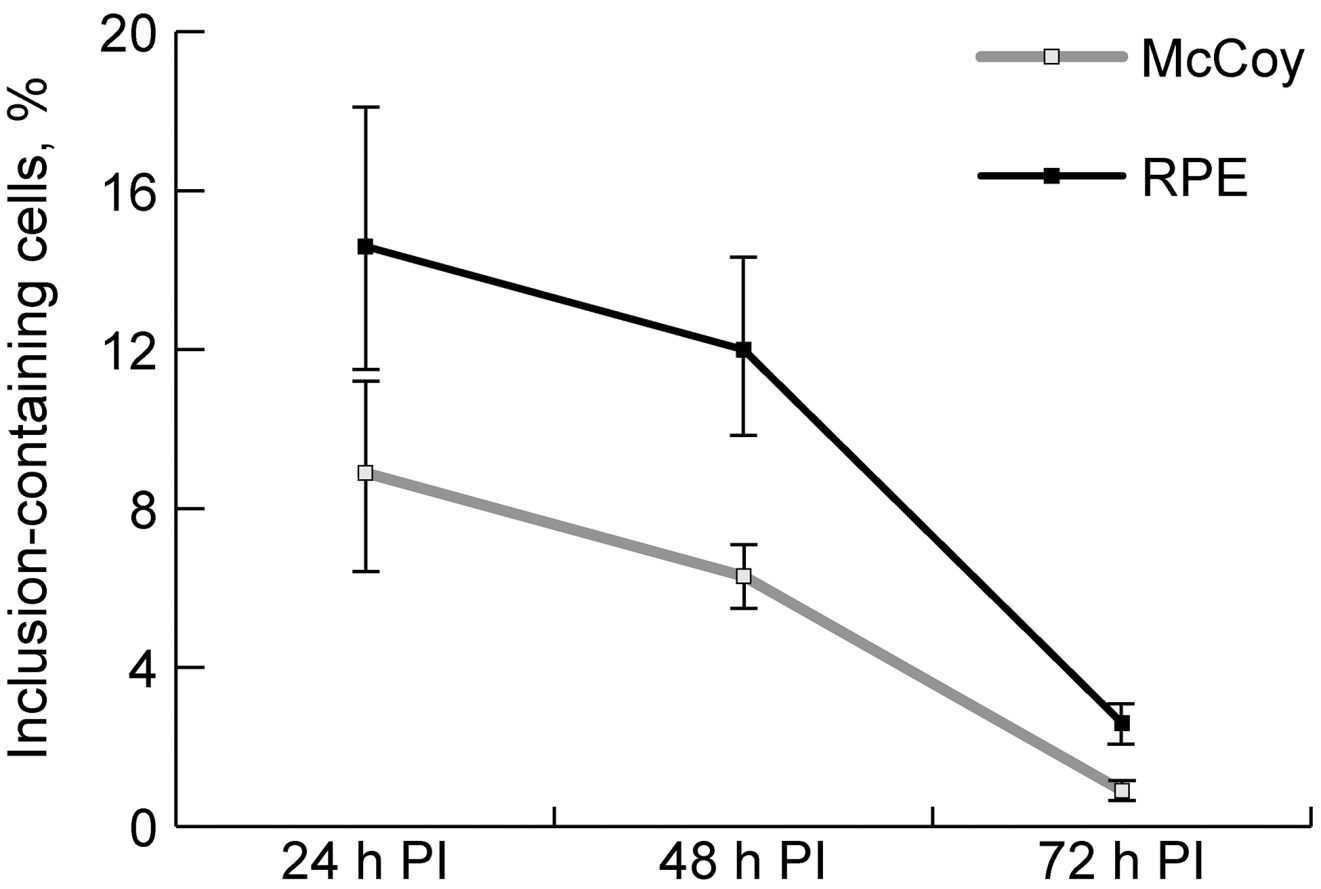
Changes in the percentage of inclusion-containing cells in hRPE and McCoy cell cultures following inoculation with *Chlamydia trachomatis* (clinical isolate No.24032).

Forty-eight hpi, the number of inclusions decreased (Fig 1) due to the release of a new generation of EBs from the largest inclusions. The appearance of cells with a broken cell membrane and release of EBs to the extracellular environment was observed as the *Chlamydia trachomatis* life cycle was approaching its completion. The released EBs were seen attached to adjacent cells. Seventy-two hpi, a massive release of the EBs from intracellular inclusions was seen in culture specimens; this corresponded to the detection of numerous inclusions exhibiting weak immune responsiveness (Fig 3A) by immunohistochemistry. Numerous EBs were seen in the microscopy projection image of cells without inclusions (likely, on cell membranes) (Fig 3B), which was not observed at 48 hpi. The progression in the reduction in the percentage of inclusion-containing cells in hRPE culture was observed in a way similar to that in McCoy culture at 72 hpi (Fig 4). Repassage of the pathogen from RPE cell culture to RPE and McCoy cell cultures at 72 hpi was followed by the formation of multiple *Chlamydia trachomatis* inclusions in both RPE and McCoy cell cultures.

### Changes in the Expression of Signaling Molecules and Proteins of Cytoskeleton and Extracellular Matrix in hRPE Cells Grown in Culture

**Fig 5:**
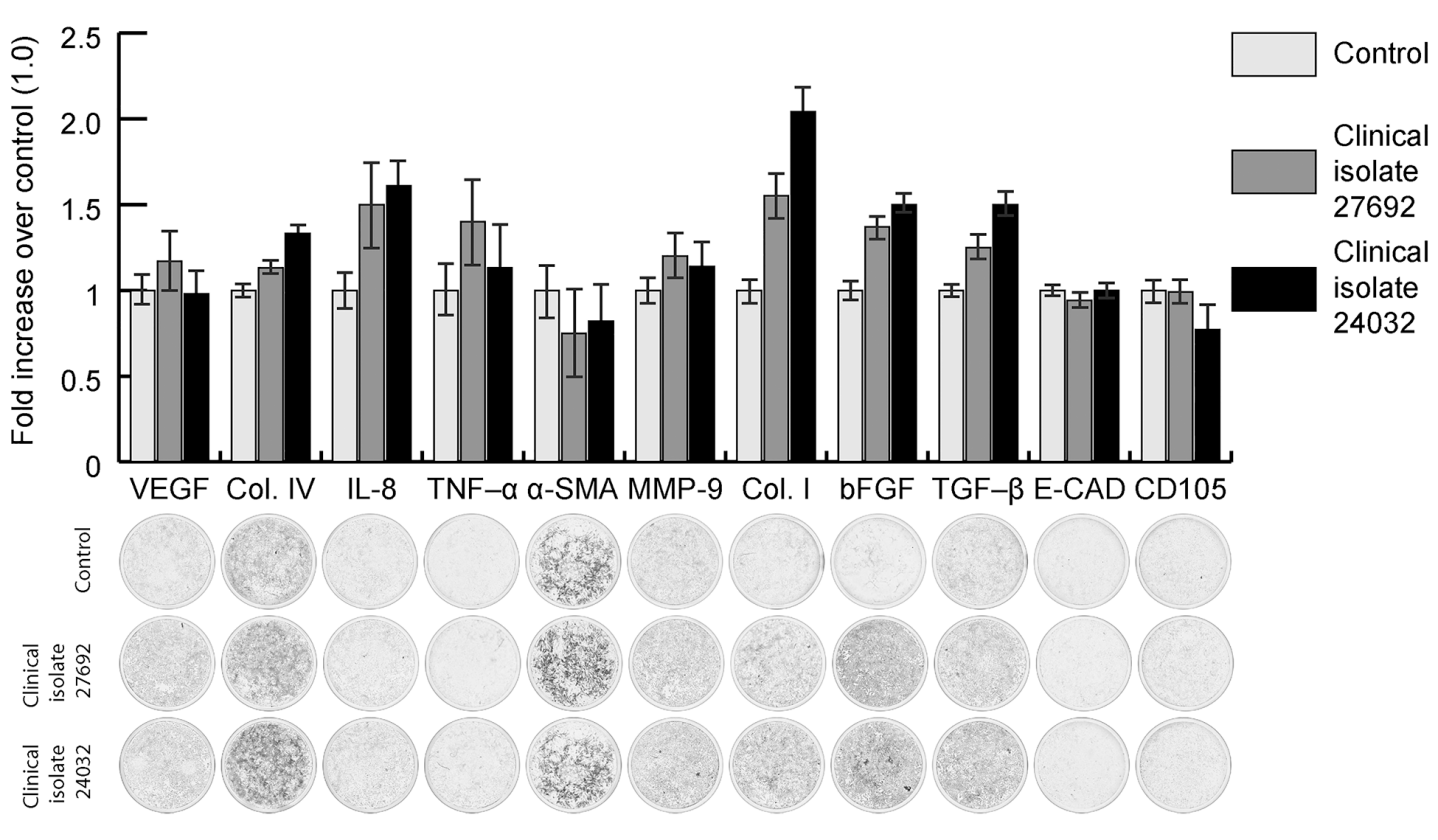
Changes in the expression of signaling molecules and proteins of cytoskeleton and extracellular matrix of hRPE cells grown in culture following inoculation with *Chlamydia trachomatis* (clinical isolates Nos. 24032 and 27692).

Statistically significant differences in the expression of collagen type IV, IL-8, collagen type I, bFGF and transforming growth factor-β (TGF–β) molecules were revealed between the *Chlamydia trachomatis*-infected and control specimens as well as between those infected with the two clinical isolates (Fig 5). Additionally matrix metalloproteinase (MMP)-9 were increased, although not statistically significantly, in the culture specimen infected with clinical isolate No.27692 compared to the control. A similarity in the expression of vascular endothelial growth factor (VEGF), tumor necrosis factors–α (TNF–α), α-smooth muscle actin (α-SMA), MMP-9, E-cadherin and СD105 was observed between the *Chlamydia trachomatis*-infected and control specimens. The number of *Chlamydia trachomatis* inclusions in the culture specimen infected with clinical isolate No.24032 was 3.28 times higher than in that infected with clinical isolate No.27692; this value was not statistically significantly different from that obtained during the assessment of infectivity of clinical isolates.

## Discussion

This study demonstrates for the first time the possibility of infecting hRPE cells with *Chlamydia trachomatis*. Infectivity of *Chlamydia trachomatis* clinical isolates for RPE culture was found to be at least as high as that for the culture commonly used for the survival and propagation of rather small numbers of *Chlamydia trachomatis* EBs. Therefore, we found, also for the first time, that the RPE can be considered as highly susceptible to *Chlamydia trachomatis* infection. It is this finding that is of primary importance in this study, since there is no data on natural resistance of specific cell cultures to infection *in vitro* with *Chlamydia trachomatis*. Because a wide variation in the percentage of inclusion-containing cells (1.5% - 14.6%) was observed between the hRPE culture specimens inoculated with different clinical isolates, this percentage is determined both by the susceptibility of the cells to infection and by the infectivity of a clinical isolate. Interestingly that isolates demonstrating higher levels of infectivity for McCoy cells showed also higher levels of infectivity for RPE cells than those with lower levels of infectivity for McCoy cells, thus evidencing for the absence of specific resistance of RPE cells to *C*. *trachomatis*.

In hRPE cultured cells, the pathogen goes through a complete life cycle, which is completed with the release of numerous new infectious EBs that are capable of maintaining a *Chlamydia trachomatis* population. This is first confirmed by the results of further repassages of the pathogen in RPE culture. Moreover, this fact is supported by (1) the similarity in the dynamics of the percentage of inclusion-containing cells in hRPE and McCoy cultured cells and (2) images of the release of EBs from intracellular inclusions, and images of multiple EBs on cell membranes of RPE cells at 72 hpi.

Pro-fibrogenic and proinflammatory changes in the expression of signaling molecules, cytoskeleton and ECM components in RPE cells following the infection with *Chlamydia trachomatis* were demonstrated by the selective increase in the expression of collagen type IV, collagen type I, IL-8, bFGF and TGF–β.

Increased expression of collagens (type I and IV) has been observed in patients with trachomatous conjunctivitis, demonstrating the capacity of *Chlamydia trachomatis* to modulate the collagen expression [13]. This agrees with the results of this study. Infection of male genital tract (in vivo and in vitro) [14], cervical [15], colonic [15] and some other [13, 16] epithelial cell cultures with *Chlamydia trachomatis* is known to upregulate secretion of IL-8. Interleukin-8 is considered to play an important role in the induction of inflammation following infection with *Chlamydia trachomatis* [17]. *Chlamydia trachomatis* infection *in vitro* has been shown to upregulate TGF-β [18], which promotes scar tissue deposition. Induction of expression of bFGF, which is crucial for remodeling connective tissue, by human smooth muscle cells has been observed following *Chlamydia trachomatis* infection [19]. In our study, upregulation of IL-8, TGF-β and bFGF was also found in RPE cells. TGF-β can increase MMP expression. In our study, the MMP-9 level in *Chlamydia trachomatis*-infected RPE culture was increased, but the value was not statistically significantly different from that of the control. This warrants further investigation of MMP expression in RPE *Chlamydia trachomatis* infection, since upregulation of MMP-9 (including that in the absence of changes in the expression of TGF-β) was demonstrated *in vitro* in porcine retinal cell line VIDO R1 [20].

We did not find significant changes in the expression of α-SMA, VEGF, E-cadherin and CD105. This might be expected, since *Chlamydia trachomatis*-induced changes in the expression of α-SMA, E-cadherin and CD105 have never been reported. In a number of works, upregulation of TNF–α expression in *Chlamydia trachomatis*-infected cell cultures has been reported [18, 21, 22], sometimes in association with arrest of the pathogen life cycle [21]. RPE culture cells inoculated with *Chlamydia trachomatis* showed no significant change in expression of TNF–α. This corresponds to a weak response, and partly explains their high susceptibility to *Chlamydia trachomatis* infection.

One could hypothesize that the increase in expression of collagen type IV, collagen type I, IL-8, TGF–β and bFGF may be partly explained by stimulation of RPE cells with *Chlamydia trachomatis* antigen. However, the *Chlamydia trachomatis* doses (and, correspondingly, the amounts of introduced antigen) used to compare the effect of two clinical isolates were identical, the difference being only the amount of intracellular inclusions formed. Because the expression was higher in specimens with increased number of inclusions, we believe that infection of the cells substantially contributes to the changes in expression of the mentioned molecules.

In vitro damage to the hRPE by West Nile virus [9], influenza A viruses [10], cytomegalovirus [23], HSV-1 [22], and HIV [24] has been described in literature. Damage to the posterior segment can occur following infection with these viruses[25–27]. The results of cultural studies are therefore in general agreement with infection prevalence in ocular tissues. Because *Chlamydia trachomatis* can spread not only by cell-to-cell contact, but also hematogenously [28], the development of intraocular infection appeared possible and further studies of *Chlamydia trachomatis* infection *in vivo* of RPE cells are required.

Given the proinflammatory and proproliferative effect of *Chlamydia trachomatis* on RPE cells, which was demonstrated by the upregulation of collagen type IV, collagen type I, IL-8, TGF and FGF, the results of the study should be extrapolated primarily to humans with proliferative vitreoretinopathy, because in this disease, production of ECM (including collagen type I) [29], TGF [30, 31], FGF [31], and IL-8 [32] is of particular importance.

The present study has several limitations. Particularly, although RPE cultures of different donors may vary in susceptibility to infection due to genetic or other individual features, the assessment of infectivity of the pathogen was performed using the source material of a single donor. Nevertheless, this was necessary to compare the infectivity of different clinical isolates. Addtionally, we assessed the expression of signaling molecules and proteins of cytoskeleton and extracellular matrix using a semi-quantitative method.

In conclusion, this study, for the first time, proved the possibility of infecting hRPE cultured cells with *Chlamydia trachomatis*, which leads to proproliferative and proinflammatory changes in the expression of signaling molecules and ECM components. Furthermore, we demonstrated (1) increased susceptibility of RPE cells to *Chlamydia trachomatis* infection, and (2) variability in infectivity and in the influence on the expression of collagen type IV, collagen type I, IL-8, TGF-β and bFGF among different clinical *Chlamydia trachomatis* isolates. These results may be of importance in studying the vitreoretinal pathology.

## Supporting Information

S1 File These are raw data from calculation of percentage of inclusion-containing cells in hRPE and McCoy cell cultures following inoculation with different Chlamydia trachomatis clinical isolates and optical density measurement of expression of signaling molecules and proteins of cytoskeleton and extracellular matrix of hRPE cells grown in culture following inoculation with Chlamydia trachomatis. (XLSX)
